# Peptide-Based Vaccination for Antibody Responses Against HIV

**DOI:** 10.3390/vaccines7030105

**Published:** 2019-09-02

**Authors:** Behazine Combadière, Manon Beaujean, Chloé Chaudesaigues, Vincent Vieillard

**Affiliations:** Sorbonne University, UPMC Univ Paris 06, INSERM, U1135, CNRS, ERL 8255, Center of Immunology and Infectious Diseases (CIMI-Paris), 91 Boulevard de l’Hôpital, F-75013 Paris, France

**Keywords:** peptide-conjugate, HIV-1, neutralizing antibodies, vaccination, adjuvants

## Abstract

HIV-1 is responsible for a global pandemic of 35 million people and continues to spread at a rate of >2 million new infections/year. It is widely acknowledged that a protective vaccine would be the most effective means to reduce HIV-1 spread and ultimately eliminate the pandemic, whereas a therapeutic vaccine might help to mitigate the clinical course of the disease and to contribute to virus eradication strategies. However, despite more than 30 years of research, we do not have a vaccine capable of protecting against HIV-1 infection or impacting on disease progression. This, in part, denotes the challenge of identifying immunogens and vaccine modalities with a reduced risk of failure in late stage development. However, progress has been made in epitope identification for the induction of broadly neutralizing antibodies. Thus, peptide-based vaccination has become one of the challenges of this decade. While some researchers reconstitute envelope protein conformation and stabilization to conserve the epitope targeted by neutralizing antibodies, others have developed strategies based on peptide-carrier vaccines with a similar goal. Here, we will review the major peptide-carrier based approaches in the vaccine field and their application and recent development in the HIV-1 field.

## 1. Introduction

Since the identification of the Human Immunodeficiency Virus type 1 (HIV-1) as the etiologic agent of AIDS (Acquired Immunodeficiency Syndrome), many efforts have been made to stop the AIDS pandemic. A major success of medical research has been the development of the highly active antiretroviral therapy. However, a safe and effective vaccine able to prevent and eradicate the HIV pandemic is still lacking and its development remains a daunting challenge that faces major obstacles.

Abundant data have shown that broadly neutralizing antibodies (bnAbs) are induced in natural HIV infection and that such bnAbs, provided by passive transfer, can both protect from HIV in robust animal models and affect ongoing HIV infection in humans [1]. This suggests that stimulation of nAbs with broad specificity for all HIV variants by a vaccine is undoubtedly the best approach [2]. However, eliciting such bnAbs by vaccination is fraught with difficulties. The envelope (Env) HIV protein is a heavily glycosylated trimeric protein consisting of three glycoprotein (gp) 120 molecules noncovalently associated with three transmembrane gp41 molecules [3]. This unique system has formidable defenses that hinder bnAbs development, linked to its conformational dynamic and extremely variable sequence. Thus, the use of HIV-1 in live/attenuated vaccine formulations and other traditional vaccine approaches was unworkable or inefficacious, whereas extraordinary progress has been made in understanding the immune response to HIV infection and in defining viral targets. We now have a detailed vision of the obstacles we face, including the discovery and characterization of potent bnAbs isolated from both infected individuals and immunized animals, the description of the structure of the Env trimer, which is the sole target of nAbs and the determination of the course of Ab-virus coevolution in individuals who develop bnAbs responses. These new parameters serve to design new immunogens and strategies for HIV bnAbs-based vaccines. 

Although many vaccine candidates are composed of the whole virus or specific proteins, the use of only a minimal pathogen epitope which can stimulate long-lasting protection is becoming a tendency in vaccine development. Peptide-based vaccines are a major focus of this field since they are easier to produce and show more stability than whole proteins. Furthermore, synthetic peptides have several benefits compared to other kinds of antigens, showing an absence of potentially damaging materials, lower antigen complexity, and low costs for scaling up. Regarding the immune response, peptide-based vaccines can generate specific responses and they can be combined to design multi-epitopes and/or multi-specific vaccines. Peptide vaccine studies, which were becoming increasingly marginalized just a few years ago, are now on the rise as a promising approach for the rational design of vaccines.

In this review, we report recent developments in the design of peptide-vaccines aimed at inducing bnAbs. We will be focused on considerations for their design and delivery, taking into account some recent peptide-vaccine candidates that are rapidly expanding this new area of research to develop a potent and affordable HIV-vaccine.

## 2. Considerations for The Design of Peptide Vaccines

A variety of considerations need to be made during the design of a peptide vaccine, in the context of the particular vaccine under development. First and foremost is the identification of immuno-dominant domains in the HIV envelope glycoproteins, which form the sole entry complex for HIV. Further major challenges are how to avoid inactivation or degradation by the immune system and how to enhance the immunogenicity of those peptides. Thus, challenges in vaccine formulation should be considered, such as antigen-carrier systems, adjuvants, the formulation, and the route of administration [4,5]. The appropriate carrier system for delivery and right formulation to produce an adjuvant effect are essential. A search in clinicaltrial.gov proposes 591 clinical studies using peptide vaccines (2019 Data Base). Yet, the design of an efficient antigen-carrier vaccine necessitates the identification of the potential correlate or surrogate of protection [6,7]. 

## 3. Strategies for Peptide Mapping

For the development of a broad-spectrum vaccine against multiple viral serotypes for maximum clinical benefits [8], it might be necessary to identify highly conserved epitopes. Epitope choice is a crucial stage in the development of a peptide vaccine; the epitopes must be able to induce strong, long-lasting bnAbs activity against HIV. These Abs target the viral entry machinery in the HIV Env protein and have characteristics that might make them difficult to elicit by vaccination, including targeting the CD4-binding site which has extensive somatic hypermutation, the trimer apex that requires unusual recombination, the glycan-V3 supersite that requires the recognition of *N*-linked glycan, and the membrane-proximal external region [9]. Infection with HIV-1 results in Ab responses to most viral proteins; however, Abs to the surface Env are the most capable of mediating virus neutralization [2], thus, the Env immunogen seems to be the most relevant target for the induction of such Abs by vaccination. However, Env has evolved a number of immune evasion strategies that create a major hurdle for vaccine design, particularly its extensive amino acid variation, structural and conformational instability, and immunodominance of hypervariable regions [3,10]. Furthermore, despite some functionally conserved requirements for viral entry, genes encoding Env display an enormous amount of diversity and can be divided into genetic subtypes A–K, also called clades. Progress in understanding bnAbs and insight into the structural characteristics of both the Env and Abs have led to ways to think about the development of bnAbs and to propose strategies for their induction. Notably, several targets for bnAbs have been characterized, including the CD4-binding site, gp120 V1-V2 glycan, V3-glycan, and the interface between gp120-Gp41, the gp41 fusion domain, and the gp41 envelope proximal external region [2].

Today, a new wave of technological breakthroughs over the past decade have potentiated the identification of new epitopes [11]. The first strategy is the in-silico approach that usually focuses on predicting B-cell epitopes. Until recently, vaccine development was associated with conventional methods such as biochemical, immunological, and microbiological approaches using the whole or part of the HIV envelope proteins. With the advent of post-genomic techniques and informatics for immune system data analysis, reverse vaccinology is becoming a useful tool to design and develop vaccines; in short, reverse vaccinology uses immunoinformatics for epitope mapping using predictive algorithms that are able to predict B-cell peptide epitopes [12]. Various studies have been performed using peptide epitope predictions for previously known immunogenic proteins. These algorithms have become more powerful in predicting epitopes and can be combined to increase the accuracy of large-scale peptide epitope predictions in HIV. For example, Bricault et al. [13] recently demonstrated that bnAbs signatures can be used to engineer HIV-1 Env vaccine immunogens capable of eliciting Ab responses with greater neutralization breadth by a machine-learning-based prediction approach [13,14]. The second strategy is in vitro toward the discovery of new epitopes by a phage display technology, resulting in the expression of a peptide that mimics the structure of an epitope. This reverse technology approach was used to select peptides that mimic HIV-1 epitopes recognized by different bnAbs such as VRC01 [15,16,17]. Alternatively, immunodominance assays and peptide competition assays are used to identify and characterize new epitopes based on antigen-presenting cells (APCs) or fluorescence-labeled peptides. 

## 4. Strategies to Increase Immunogenicity

Viruses and other natural pathogens are recognized by the innate defense systems as a danger signal and alert the immune system of the presence of invaders. Antigen-presenting cells (APCs), such as dendritic cells (DCs) or macrophages, are able to recognize pathogen-associated molecular patterns (PAMPs) via pattern recognition receptors (PRRs), such as toll-like receptors (TLRs) [18]. Clearly, these motifs are missing in peptide-based vaccines, which necessitates adjuvants for the stimulation of innate cells for the induction of adaptive immunity. Peptides are poorly taken up by antigen-presenting cells (APC) via MHC molecules and thus result in the inefficient priming of CD4^+^ T cells, with consequently a low production of Abs. To bypass this side-effect, peptide-based vaccines require a carrier to be presented, as well as efficient adjuvants and an appropriate delivery system to be effective.

In the late 1920′s, Landsteiner, Avery, and Goebel showed that the immunogenicity of polysaccharides could be enhanced by coupling to a protein. Five major carrier proteins have been used in licensed conjugate vaccines: A genetically modified Cross-Reacting Material (CRM) of the diphtheria toxin (DT), the tetanus toxoid (TT), the meningococcal outer membrane protein complex (OMPC), the diphtheria toxoid, and *Haemophilus influenzae* protein D (HiD) [19]. Several parameters need to be considered: (1) Small molecules need to be preserved from degradation and the initial conformation of the epitope needs to be conserved for B cell responses (formulation, adjuvants), (2) there needs to be a bridge to T cell help (carrier addition), and (3) the peptide-epitope might not have conserved the initial conformation for B cell responses (linker to carrier). Some modifications are thus necessary to stabilize the peptide conformation. For example, flanking sequences at the C and N terminus “cassette” would help in conformational stabilization within alpha-helical coiled coil proteins [20]. This approach has been developed and applied to two proteins: The Streptococcal M protein and *Caenorhabditis elegans* para-myosin UNCoordinated-15 (UNC-15) protein for antigenic B cell epitopes. In some cases, an artificial bond between two side chains of amino acid could be envisaged. Bird et al. [21] explored the capacity of peptide stapling to generate high fidelity, protease-resistant mimics of antigenic structures for HIV-1 gp41 epitopes. Stabilized α-helices of the membrane proximal external region (SAH-MPER) of gp41 were shown to be protease-resistant and bound to the broadly neutralizing 4E10 and 10E8 Abs with high affinity [22]. However, these methods need to be used carefully; increasing or decreasing the affinity of epitopes to Abs might suppress their immunogenicity and induce tolerance. Epitope stapling and peptide cyclisation has been a popular strategy in HIV vaccine development. Different metabolic additions could help to stabilize the peptide, including amidation of the C-terminus and carbohydrate or lipid addition [11,21,22,23]. To target APCs, peptides could be coupled to mannose or glycan that bind to C-lectin present at the cell surface of DCs and macrophages [24]. In addition, some designs of vaccine candidates have given careful consideration to coupling the peptide with a plasma membrane transporter protein to facilitate its penetration into the cell. The most popular was a peptide of the Tat protein from HIV-1 that passes through the cell membrane [25]. To increase the up take by cells, the Tat peptide was coupled with several other peptides. This peptide was also used in combination with the amphipathic alpha-helical antitumor peptide (HPRP-A1) to increase the rate of cellular uptake and allow a stronger antitumor activity [26]. 

The delivery system is another major challenge in peptide vaccine development to induce the correct cellular cascade for the production of bnAbs. Proper targeting of small antigens, such as peptides to APC, lead to T helper (Th) cells for B cell activation and the production of Abs [27]. Several populations of dendritic cells (DCs) have been shown to control T Follicular Helper (Tfh) induction and, thus, B-cell activation and Ab responses. Depending on the route of immunization, myeloid DCs, conventional DCs, and Langerhans cells in the skin might control Tfh induction in humoral responses [28,29,30,31,32]. Activation of Tfh cells thus has a pivotal role in B cell differentiation and the formation of germinal centers (GCs) in lymphoid tissue [33]; they are essential for GC maintenance and for affinity maturation. The concept of antigen-carrier system delivery also includes the solicitation of both T and B cell responses and the induction of a memory response to the vaccine for long-term protection. There is an extensive variety of adjuvants that show in vitro efficacy in the induction of immune responses against peptides. The first approved vaccine adjuvant was aluminum hydroxide (Alum); it was developed by Glenny et al. in 1926 with the diphtheria toxoid absorbed to Alum, and used for over eight decades [34]. However, Alum poorly absorbs peptides and might expose them to proteolytic degradation, suggesting that the peptides might lose their potency to induce protective immune responses. In 1936, Freund developed an emulsion of water and mineral oil including killed mycobacteria, known as Freund’s adjuvant, a gold standard adjuvant for efficacy, however, it induces severe local necrotic ulcers and is thus considered as too toxic for human use [35]. Fortunately, several new adjuvants were approved by the FDA and Europe for human vaccines, such as MF59, virosome, AS03, AF03, and AS04 (monphosphoryl lipid A (MPL) with Alum). MF59 is composed of squalene and is produced by Novartis Vaccines and Diagnostics Inc. It is used as an adjuvant component of the influenza vaccine for elderly patients (Fluad^®^, Novartis Vaccines and Diagnostics, Cambridge, MA, USA) [18,36]. It has also been shown that an oil in water emulsion provides a slow release of a peptide-based cancer vaccine for sustained antigenic stimulation of adaptive immunity [37]. The potency of the emulsion could depend also on the surfactant. Indeed, montanide TM ISA51 includes mono-oleate in addition to the squalene emulsion and has been used for melanoma peptide formulation in a clinical trial [38,39]. Interestingly, some adjuvants can be used in combination; several examples have been already approved by the FDA, such as Cervarix, a vaccine from GSK Biologicals (London, United Kingdom), composed of the TLR4 agonist monophosphoryl lipid A (MPLA) together with Alum.

The peptide-carrier or peptide alone can also be trapped in liposomes or covalently linked to liposomes to be delivered. Their composition of an aqueous core and lipid bilayer outside has the advantage of mimicking cell membranes [8,40], and thus, is biocompatible and adapted to delivery to APCs. As an example, the influenza peptide (H3N2 NP366–374) conjugated with an oleyl liposome protects against an influenza virus challenge. It is important to note that modification of the liposome size, charge, and injection routes might modify the quality of the immunological outcome, such as T_H1_ versus T_H2_ responses [41,42,43]. Some studies have shown that pH sensitive liposomes release their contents into the cytosol after fusion with the endosomal membrane and promote CTL responses. Such studies were conducted with the HPV, peptide, or HIV peptides [44,45].

## 5. Peptide-Based Vaccination for Antibody Responses against HIV

Substantial efforts have been made to create a stabilized Env trimer that elicit Abs potently effective against a broad range of HIV-1 circulating strains [10,46,47]. A parallel approach that relies on capturing Env sequences that have elicited bnAbs in infected individuals has also been developed. A unique patient cohort was mined to select and express soluble Env immunogens from patient sequences most relevant to viral transmission, including Env from founder viruses of diverse clades, and from “elite neutralizers” [48]. Of note, the majority of identified bnAbs have characteristics that make them difficult to elicit by vaccination since extensive somatic hypermutation appears to be required. These features reflect a long and intensive selection process from which high affinity B cells are generated in the germinal center [33,49].

Even though the focus of HIV vaccine studies has been on the Env protein in its trimeric form, other approaches defining precise target of Abs combined with structural biology have provided evidence for an epitope-based vaccine candidate. In addition, to overcome the viral diversity of HIV-1, most of the strategies drive the Ab response towards the more conserved epitopes of Env. The major peptide-vaccines are reported in Table 1. Several are HIV-specific peptide epitopes selected by screening random libraries with HIV-1 positive sera [50]. Among these different peptide-vaccines, Palker et al. [51] described some Env-encoded synthetic peptides coupled to the tetanus toxoid that raised high titers of isolate-specific anti-HIV bnAbs. These peptides were selected from phage libraries with plasma Abs from HIV-infected Long-Term Non-Progressor (LNTP) patients. The mimotope sequences were analyzed for homology to HIV-1 Env, in particular for their capacity to represent conformational epitopes on the surface of the gp120 structure, and then coupled to a sequential oligopeptide carrier (SOC) and palmitic acid for vaccination. Their capacity to induce bnAbs was confirmed in preclinical models [51]. 

Although in theory high-affinity Abs that recognize the relatively conserved Gly-Pro-Gly-Arg (GPGR) core sequence of the HIV-1 Env V3 tip region could potentially neutralize many strains of the HIV-1 clade B, in practice, such Abs in sera from HIV-infected individuals show little neutralization activity in vitro, suggesting that the immunogenicity of the GPGR sequence is low. To overcome this problem, Eda et al. [54] sequentially immunized mice with V3 peptides from HIV-1 clade B field isolates, resulting in the induction of cross-neutralizing Abs against primary isolates. Alternatively, by a bioinformatics approach, an innovative multivalent HIV-1 vaccine was developed, comprising of a pool of 176 peptides representing variable regions of the Env and Gag proteins. In macaques, a neutralizing activity against 14 HIV-1 primary isolates from subtypes A–E was observed in three to six macaques, according to in vitro assays [52]. This was the first time that bnAbs had been demonstrated against HIV-1 isolates using linear hypervariable epitopes as immunogens, however, the potential efficacy of this strategy against an SIV challenge is still unclear.

Another peptide of 29 amino acids that overlaps with the epitope of the first generation 2F5 mAb was also identified with the aim of eliciting bnAbs upon vaccination. After optimization, the EC26-2A4 peptide was tested for its immunogenicity and potency to induce bnAbs in a preclinical model [55]. The design of a peptide carbohydrate is another approach to explore novel vaccine candidates to elicit neutralizing antigens, based on a peptide DP178 (mimotope) that inhibits the viral fusion of HIV-1 and is recognized by 2F5 bnAbs. Enhancement of a-helicity in the DP178 leads to an increased affinity to 2F5 without eliciting bnAbs [56]. However, the breath of these bnAbs is still questionable. 

Recently, Kwong and colleagues [61] described a vaccination scheme based on the fusion peptide, a critical element of the HIV-1 entry machinery. The fusion peptide comprises a hydrophobic region (residues 512–527) at the N terminus of the gp41 transmembrane subunit of the HIV-1 Env trimer. They identified an Ab N123-VRC34.01 from a HIV+ donor that targets the conserved N-terminal region of the HIV-1 fusion peptide. Immunization with this FP8 peptide (residues 512–519) coupled to keyhole limpet hemocyanin (KLH), followed by Env trimer boosts, elicited cross-clade neutralizing responses [61]. In mice, the FP8 elicited monoclonal Abs capable of neutralizing 31% of a cross-clade panel of 208 HIV-1 strains. In guinea pigs and rhesus macaques, a serum neutralization breadth of 20% or higher-as assessed across a panel of 208 isolates-was only observed sporadically [61]. Consistent elicitation of cross-clade HIV-neutralizing responses were, however, achieved in guinea pigs by repetitive envelope trimer boosting [62]. More recent data of cryo-electron microscopy of vaccine-elicited Abs in complex with Env trimer revealed focused recognition of the fusion peptide and provided a structural basis for development of neutralization breadth, suggesting that the breadth of vaccine-elicited antibodies targeting the fusion peptide can be enhanced by specific interactions with additional portions of Env [63,64]. 

Another promising peptide of gp41 has been identified as a potential vaccine candidate [56]. The 3S motif of gp41 is located in the gp41 loop region (according to the position of gp160 of the BRU HIV-1 strain) and is remarkably conserved and specific to HIV-1 strains. This epitope is located at the interface between the cell membrane and HIV-1 Env protein (Vieillard et al., personal communication). These features indicate that this motif is probably important for the conformation and/or the functionality of the virus [65]. Natural anti-3S Abs have been found in several cohorts of HIV patients and are related to disease parameters. In HIV progressors, anti-3S Abs are detected in 28% of patients and are highly correlated with CD4^+^ T cell counts. In addition, in the SEROCO cohort of seroconvertors (French National Agency for Research on AIDS; ANRS), a high level of anti-3S Abs delayed spontaneous disease progression independently of viral load and CD4 count. Of note, natural anti-3S Abs detected in HIV patients were non-neutralizing. From these data, the VAC-3S therapeutic vaccine was developed, comprising the 3S peptide coupled with the CRM197 carrier protein formulated in an aluminum salt adjuvant (licenced by Minka Therapeutics). Moreover, a multi-center phase 2, randomized, double-blind, placebo-controlled clinical study revealed that VAC-3S is safe, immunogenic, and associated in high responders with an increase in non-exhausted CD4^+^ T cells [58].

The unique properties of the 3S peptide were further studied by alanine-scanning of the 3S motif. A specific substitution at position 614 (called W614A-3S) is able to elicit neutralizing Abs in mice with the capacity to block viral infection [60]. These data demonstrate that W614A-3S is antigenic and that natural anti-W614A-3S Abs were also observed in approximately 5% of HIV-1 patients with high CD4 counts. These natural Abs were immunoglobulin G isotypes and detected exclusively in patients with high CD4 counts and undetectable viral loads (<20 copies/mL). The W614A-3S purified Abs from most patients were able to neutralize high titers of all tier 1 (clade B and C) and most of tier 2 viruses of clade B tested. Strikingly, purified Abs from one of these five patients was also reactive against tier 2 clade C (PSV-TV1) and clade E (PSV-CM244) viruses. No W614A-3S purified Abs neutralized HIV-2 ROD or VSV, which were used as negative controls [60]. Interestingly, the presence of anti-W614A-3S nAbs was rarely observed in HIV-1 progressors but was significantly increased in untreated LTNP patients of the ALT ANRS cohort that had been infected more than 7 years but did not declare the disease. The presence of W614A-3S Abs was inversely correlated with viral load and viral DNA and associated with preservation of the CD4 count, however, these Abs were also found to be able to neutralize most of the HIV-1 clade B viruses tested [66], providing a strong argument that W614A-3S nAbs might contribute to the LTNP status. In order to test the immunogenicity of the W614A-3S motif, two different carrier proteins have been used: KLH (preclinical studies only) and CRM-197 (preclinical and proposed clinical studies). W614A-3S-KLH in Incomplete Freund Adjuvant was shown to be immunogenic in a mouse model and induced Ab responses with cross-clade neutralizing activities [60]. This effect has also been shown in macaque cynomolgus and in rabbits (Vieillard et al., personal communication). Translation of this new peptide-vaccine candidate into an experimental medicine study is planned for 2020 and will speed up progress in HIV-1 vaccine development. 

An important question concerns whether animal models need to be used to test these peptide-based vaccine candidates. The vast majority of HIV research has been performed using either humanized mice or NHP HIV models. Under the umbrella terms “NHP” and “humanized mice” are a myriad of noteworthy model characteristics [66]. Although no perfect animal model for human disease exists, each of these systems has advantages and disadvantages for assessing new bnAbs that target the HIV-1 Env protein in a peptide-vaccine strategy. The question remains of whether or not there should be a standardized mode. Differences in SIV and SHIV replication in the rhesus macaque, cynomolgous, and pigtailed macaque species have been observed and favor the design of experimental models depending on the question asked. For vaccine research, the rhesus macaque presents the ideal model for pathogenesis research, however, demanding its use as a standard presents problem, and the current trend is to base considerations on transgenic mouse models. Most of the recent animal model research has focused on evaluating vaccine candidates that might elicit bnAbs.

In conclusion, due to the enormous progress in GMP production, and the safety and immunogenicity of several peptide-carrier vaccines against infectious diseases, “epitope-based vaccine” discovery has become one of the challenges of this decade and has gained interest in the field of HIV-vaccine development. Studying the structure of the envelop protein conformation and how to conserve the epitope that could be targeted by natural bnAbs will help in the development of novel peptide-vaccine candidates. 

With regard to the Thai RV144 trial, the generation of partially protective non-neutralizing Abs questions the role of the other Ab functions, such as Ab-dependent cellular cytotoxicity, Ab-dependent cellular phagocytosis, or aggregation [67]. These other functions of Abs should be also considered and used to predict vaccine efficacy. Finally, implementation of more clinical trials with peptide-vaccines should foster research in the development of a safe and effective vaccine against HIV.

## Figures and Tables

**Table 1:** List of peptide discovery-based HIV vaccine candidates inducing non neutralizing and neutralizing antibody responses.

Epitope	Carrier	Pre-Clinical Test	Humoral	Translation into Clinical Studies	Ref.
Peptide screening library		Mice	bnAbs	none	[50]
		Macaques	Reduction viremia below the levels of detection	none	[52,53]
V3 peptides		Mice	bnAbs	none	[54]
EC26-2A4	SOC*/palmitoyl acid	Mice	bnAbs	none	[53]
	KLH	Mice	bnAbs	none	[55]
DP178 and structured analogs	KLH	Guinea pigs	Abs	none	[56]
3S motif	KLH	Macaques	Prevented a decline in CD4^+^ T cells		[53,57]
	CRM197	Human	Significant increase in CD4^+^ T cellsNatural bnAb detected in HIV-1^+^ patients	Clinical study Phase II	[58,59]
W614A 3S motif	KLH	MiceHuman	bnAbs Natural bnAb detected in HIV-1^+^ patients (LTNP)	Planned for 2020	[59,60]
Fusion Peptide (FP)	KLH	Mice, Guinea pigNon-human Primates	bnAbs	none	[61,62]

* SOC: oligopeptide carrier.

## References

[1] Caskey M., Klein F., Nussenzweig M.C. (2019). Broadly neutralizing anti-HIV-1 monoclonal antibodies in the clinic. Nat. Med..

[2] Burton D.R., Hangartner L. (2016). Broadly Neutralizing Antibodies to HIV and Their Role in Vaccine Design. Annu. Rev. Immunol..

[3] Falkowska E., Le K.M., Ramos A., Doores K.J., Lee J.H., Blattner C., Ramirez A., Derking R., Van Gils M.J., Liang C.-H. (2014). Broadly Neutralizing HIV Antibodies Define a Glycan-Dependent Epitope on the Prefusion Conformation of gp41 on Cleaved Envelope Trimers. Immun..

[4] De Gregorio E., Rappuoli R. (2014). From empiricism to rational design: A personal perspective of the evolution of vaccine development. Nat. Rev. Immunol..

[5] Rappuoli R., Santoni A., Mantovani A. (2018). Vaccines: An achievement of civilization, a human right, our health insurance for the future. J. Exp. Med..

[6] Heeney J.L., A Plotkin S. (2006). Immunological correlates of protection from HIV infection and disease. Nat. Immunol..

[7] Plotkin S.A. (2005). Vaccines: Past, present and future. Nat. Med..

[8] Matyas G.R., Mayorov A.V., Rice K.C., Jacobson A.E., Cheng K., Iyer M.R., Li F., Beck Z., Janda K.D., Alving C.R. (2013). Liposomes Containing Monophosphoryl Lipid A: A Potent Adjuvant System For Inducing Antibodies To Heroin Hapten Analogs. Vaccine.

[9] Sok D., Burton D.R. (2018). Recent progress in broadly neutralizing antibodies to HIV. Nat. Immunol..

[10] Klasse P.J., Ketas T.J., Cottrell C.A., Ozorowski G., Debnath G., Camara D., Francomano E., Pugach P., Ringe R.P., Labranche C.C. (2018). Epitopes for neutralizing antibodies induced by HIV-1 envelope glycoprotein BG505 SOSIP trimers in rabbits and macaques. PLoS Pathog..

[11] Kwong P.D., Mascola J.R. (2018). HIV-1 Vaccines Based on Antibody Identification, B Cell Ontogeny, and Epitope Structure. Immun..

[12] Rappuoli R., Bottomley M.J., D’Oro U., Finco O., De Gregorio E. (2016). Reverse vaccinology 2.0: Human immunology instructs vaccine antigen design. J. Exp. Med..

[13] Bricault C.A., Yusim K., Seaman M.S., Yoon H., Theiler J., Giorgi E.E., Wagh K., Theiler M., Hraber P., Macke J.P. (2019). HIV-1 Neutralizing Antibody Signatures and Application to Epitope-Targeted Vaccine Design. Cell Host Microbe.

[14] Magaret C.A., Benkeser D.C., Williamson B.D., Borate B.R., Carpp L.N., Georgiev I.S., Setliff I., Dingens A.S., Simon N., Carone M. (2019). Prediction of VRC01 neutralization sensitivity by HIV-1 gp160 sequence features. PLoS Comput. Biol..

[15] Delhalle S., Schmit J.C., Chevigné A. (2012). Phages and HIV-1: From display to interplay. Int. J. Mol. Sci..

[16] Chikaev A.N., Bakulina A.Y., Burdick R.C., Karpenko L.I., Pathak V.K., Ilyichev A.A. (2015). Selection of Peptide Mimics of HIV-1 Epitope Recognized by Neutralizing Antibody VRC01. PLoS ONE.

[17] Dorgham K., Pietrancosta N., Affoune A., Lucar O., Bouceba T., Chardonnet S., Pionneau C., Piesse C., Sterlin D., Guardado-Calvo P. (2019). Reverse Immunology Approach to Define a New HIV-gp41-Neutralizing Epitope. J. Immunol. Res..

[18] Del Giudice G., Rappuoli R., Didierlaurent A.M. (2018). Correlates of adjuvanticity: A review on adjuvants in licensed vaccines. Semin. Immunol..

[19] Pichichero M. (2013). Protein carriers of conjugate vaccines: Characteristics, development and clinical trials. Hum. Vaccines Immunother..

[20] Cooper J.A., Hayman W., Reed C., Kagawa H., Good M.F., Saul A. (1997). Mapping of conformational B cell epitopes within alpha-helical coiled coil proteins. Mol. Immunol..

[21] Bird G.H., Irimia A., Ofek G., Kwong P.D., Wilson I.A., Walensky L.D. (2014). Stapled HIV-1 Peptides Recapitulate Antigenic Structures and Engage Broadly Neutralizing Antibodies. Nat. Struct. Mol. Biol..

[22] McGaughey G.B., Citron M., Danzeisen R.C., Freidinger R.M., Garsky V.M., Hurni W.M., Joyce J.G., Liang X., Miller M., Shiver J. (2003). HIV-1 Vaccine Development: Constrained Peptide Immunogens Show Improved Binding to the Anti-HIV-1 gp41 MAb. Biochem..

[23] Cardoso R.M., Brunel F.M., Ferguson S., Zwick M., Burton D.R., Dawson P.E., Wilson I.A. (2007). Structural Basis of Enhanced Binding of Extended and Helically Constrained Peptide Epitopes of the Broadly Neutralizing HIV-1 Antibody 4E10. J. Mol. Biol..

[24] Sheng K.-C., Kalkanidis M., Pouniotis D.S., Esparon S., Tang C.K., Apostolopoulos V., Pietersz G.A. (2008). Delivery of antigen using a novel mannosylated dendrimer potentiates immunogenicityin vitro andin vivo. Eur. J. Immunol..

[25] Frankel A.D., Pabo C.O. (1988). Cellular uptake of the tat protein from human immunodeficiency virus. Cell.

[26] Hao X., Yan Q., Zhao J., Wang W., Huang Y., Chen Y. (2015). TAT Modification of Alpha-Helical Anticancer Peptides to Improve Specificity and Efficacy. PLoS ONE.

[27] Boscardin S.B., Hafalla J.C., Masilamani R.F., Kamphorst A.O., Zebroski H.A., Rai U., Morrot A., Zavala F., Steinman R.M., Nussenzweig R.S. (2006). Antigen targeting to dendritic cells elicits long-lived T cell help for antibody responses. J. Exp. Med..

[28] Bonifaz L.C., Bonnyay D.P., Charalambous A., Darguste D.I., Fujii S.-I., Soares H., Brimnes M.K., Moltedo B., Moran T.M., Steinman R.M. (2004). In Vivo Targeting of Antigens to Maturing Dendritic Cells via the DEC-205 Receptor Improves T Cell Vaccination. J. Exp. Med..

[29] Levin C., Perrin H., Combadiere B. (2015). Tailored immunity by skin antigen-presenting cells. Hum. Vaccines Immunother..

[30] Levin C., Bonduelle O., Nuttens C., Primard C., Verrier B., Boissonnas A., Combadière B. (2017). Critical Role for Skin-Derived Migratory DCs and Langerhans Cells in TFH and GC Responses after Intradermal Immunization. J. Invest. Dermatol..

[31] Pratama A., Vinuesa C.G. (2013). Control of TFH cell numbers: Why and how?. Immunol. Cell Biol..

[32] Vardam T., Anandasabapathy N. (2017). Langerhans Cells Orchestrate TFH-Dependent Humoral Immunity. J. Investig. Dermatol..

[33] Gitlin A.D., Mayer C.T., Oliveira T.Y., Shulman Z., Jones M.J., Koren A., Nussenzweig M.C. (2015). T cell help controls the speed of the cell cycle in germinal center B cells. Sci..

[34] Glenny A.T., Pope C.G., Waddington H., Wallace U. (1926). Immunological notes: XVII−XXIV. J. Pathol. Bacteriol..

[35] Awate S., Babiuk L.A., Mutwiri G. (2013). Mechanisms of Action of Adjuvants. Front. Immunol..

[36] Coffman R.L., Sher A., Seder R.A. (2010). Vaccine Adjuvants: Putting Innate Immunity to Work. Immun..

[37] Iseki K., Matsunaga H., Komatsu N., Suekane S., Noguchi M., Itoh K., Yamada A. (2010). Evaluation of a new oil adjuvant for use in peptide-based cancer vaccination. Cancer Sci..

[38] Nagata T., Toyota T., Ishigaki H., Ichihashi T., Kajino K., Kashima Y., Itoh Y., Mori M., Oda H., Yamamura H. (2007). Peptides coupled to the surface of a kind of liposome protect infection of influenza viruses. Vaccine.

[39] Slingluff C.L., Yamshchikov G., Neese P., Galavotti H., Eastham S., Engelhard V.H., Kittlesen D., Deacon D., Hibbitts S., Grosh W.W. (2001). Phase I trial of a melanoma vaccine with gp100 (280–288) peptide and tetanus helper peptide in adjuvant: Immunologic and clinical outcomes. Clin. Cancer Res..

[40] Alving C.R., Peachman K.K., Rao M., Reed S.G. (2012). Adjuvants for human vaccines. Curr. Opin. Immunol..

[41] Mann J.F., Shakir E., Carter K.C., Mullen A.B., Alexander J., Ferro V.A. (2009). Lipid vesicle size of an oral influenza vaccine delivery vehicle influences the Th1/Th2 bias in the immune response and protection against infection. Vaccine.

[42] Bachmann M.F., Jennings G.T. (2010). Vaccine delivery: A matter of size, geometry, kinetics and molecular patterns. Nat. Rev. Immunol..

[43] Leroux-Roels G., Van Belle P., Vandepapelière P., Horsmans Y., Janssens M., Carletti I., Garçon N., Wettendorff M., Van Mechelen M. (2015). Vaccine Adjuvant Systems containing monophosphoryl lipid A and QS-21 induce strong humoral and cellular immune responses against hepatitis B surface antigen which persist for at least 4 years after vaccination. Vaccine.

[44] Chang J.-S., Choi M.-J., Cheong H.-S., Kim K. (2001). Development of Th1-mediated CD8^+^ effector T cells by vaccination with epitope peptides encapsulated in pH-sensitive liposomes. Vaccine.

[45] Chang J.-S., Choi M.-J., Kim T.-Y., Cho S.Y., Cheong H.-S. (1999). Immunogenicity of synthetic HIV-1 V3 loop peptides by MPL adjuvanted pH-sensitive liposomes. Vaccine.

[46] Pauthner M., Havenar-Daughton C., Sok D., Nkolola J.P., Bastidas R., Boopathy A.V., Carnathan D.G., Chandrashekar A., Cirelli K.M., Cottrell C.A. (2017). Elicitation of Robust Tier 2 Neutralizing Antibody Responses in Nonhuman Primates by HIV Envelope Trimer Immunization Using Optimized Approaches. Immun..

[47] Aldon Y., McKay P.F., Allen J., Ozorowski G., Felfödiné Lévai R., Tolazzi M., Rogers P., He L., de Val N., Fábián K. (2018). Rational Design of DNA-Expressed Stabilized Native-Like HIV-1 Envelope Trimers. Cell Rep..

[48] van Gils M.J., van den Kerkhof T.L.G.M., Ozorowski G., Cottrell C.A., Sok D., Pauthner M., Pallesen J., de Val N., Yasmeen A., de Taeye S.W. (2017). An HIV-1 antibody from an elite neutralizer implicates the fusion peptide as a site of vulnerability. Nat. Microbiol..

[49] McHeyzer-Williams L.J., Milpied P.J., Okitsu S.L., McHeyzer-Williams M.G. (2015). Class-switched memory B cells remodel BCRs within secondary germinal centers. Nat. Immunol..

[50] Scala G., Chen X., Liu W., Telles J.N., Cohen O.J., Vaccarezza M., Igarashi T., Fauci A.S. (1999). Selection of HIV-specific immunogenic epitopes by screening random peptide libraries with HIV-1-positive sera. J. Immunol..

[51] Palker T.J., Clark M.E., Langlois A.J., Matthews T.J., Weinhold K.J., Randall R.R., Bolognesi D.P., Haynes B.F. (1988). Type-specific neutralization of the human immunodeficiency virus with antibodies to env-encoded synthetic peptides. Proc. Natl. Acad. Sci..

[52] Azizi A., Anderson D.E., Torres J.V., Ogrel A., Ghorbani M., Soare C., Sandstrom P., Fournier J., Diaz-Mitoma F. (2008). Induction of broad cross-subtype-specific HIV-1 immune responses by a novel multivalent HIV-1 peptide vaccine in cynomolgus macaques. J. Immunol..

[53] Chen X., Scala G., Quinto I., Liu W., Chun T.-W., Justement J.S., Cohen O.J., VanCott T.C., Iwanicki M., Lewis M.G. (2001). Protection of rhesus macaques against disease progression from pathogenic SHIV-89.6PD by vaccination with phage-displayed HIV-1 epitopes. Nat. Med..

[54] Eda Y., Takizawa M., Murakami T., Maeda H., Kimachi K., Yonemura H., Koyanagi S., Shiosaki K., Higuchi H., Makizumi K. (2006). Sequential Immunization with V3 Peptides from Primary Human Immunodeficiency Virus Type 1 Produces Cross-Neutralizing Antibodies against Primary Isolates with a Matching Narrow-Neutralization Sequence Motif. J. Virol..

[55] Ringel O., Müller K., Koch J., Brill B., Wolf T., Stephan C., Vieillard V., Debré P., Dietrich U. (2018). Optimization of the EC26-2A4 Epitope in the gp41 Membrane Proximal External Region Targeted by Neutralizing Antibodies from an Elite Controller. AIDS Res. Hum. Retroviruses.

[56] Joyce J.G., Hurni W.M., Bogusky M.J., Garsky V.M., Liang X., Citron M.P., Danzeisen R.C., Miller M.D., Shiver J.W., Keller P.M. (2002). Enhancement of α-Helicity in the HIV-1 Inhibitory Peptide DP178 Leads to an Increased Affinity for Human Monoclonal Antibody 2F5 but Does Not Elicit Neutralizing Responses in Vitro: IMPLICATIONS FOR VACCINE DESIGN. J. Biol. Chem..

[57] Vieillard V., Dereuddre-Bosquet N., Mangeot-Méderlé I., Le Grand R., Debré P. (2012). An HIVgp41 vaccine protects CD4 central memory T cells in SHIV-infected macaques. Vaccine.

[58] Vieillard V., Combadière B., Tubiana R., Launay O., Pialoux G., Cotte L., Girard P.-M., Simon A., Dudoit Y., Reynes J. (2019). HIV therapeutic vaccine enhances non-exhausted CD4^+^ T cells in a randomised phase 2 trial. Npj Vaccines.

[59] Lucar O., Su B., Potard V., Samri A., Autran B., Moog C., Debré P., Vieillard V. (2017). Neutralizing Antibodies Against a Specific Human Immunodeficiency Virus gp41 Epitope are Associated with Long-term Non-progressor Status. EBioMedicine.

[60] Petitdemange C., Achour A., Dispinseri S., Malet I., Sennepin A., Fang R.H.T., Crouzet J., Marcelin A.-G., Calvez V., Scarlatti G. (2013). A Single Amino-Acid Change in a Highly Conserved Motif of gp41 Elicits HIV-1 Neutralization and Protects Against CD4 Depletion. Clin. Infect. Dis..

[61] Xu K., Acharya P., Kong R., Cheng C., Chuang G.-Y., Liu K., Louder M.K., O’Dell S., Rawi R., Sastry M. (2018). Epitope-based vaccine design yields fusion peptide-directed antibodies that neutralize diverse strains of HIV-1. Nat. Med..

[62] Cheng C., Xu K., Kong R., Chuang G.-Y., Corrigan A.R., Geng H., Hill K.R., Jafari A.J., O’Dell S., Ou L. (2019). Consistent elicitation of cross-clade HIV-neutralizing responses achieved in guinea pigs after fusion peptide priming by repetitive envelope trimer boosting. PLoS ONE.

[63] Dingens A.S., Acharya P., Haddox H.K., Rawi R., Xu K., Chuang G.-Y., Wei H., Zhang B., Mascola J.R., Carragher B. (2018). Complete functional mapping of infection- and vaccine-elicited antibodies against the fusion peptide of HIV. PLoS Pathog..

[64] Kong R., Duan H., Sheng Z., Xu K., Acharya P., Chen X., Cheng C., Dingens A.S., Gorman J., Sastry M. (2019). Antibody Lineages with Vaccine-Induced Antigen-Binding Hotspots Develop Broad HIV Neutralization. Cell.

[65] Vieillard V., Strominger J.L., Debre P. (2005). NK cytotoxicity against CD4^+^ T cells during HIV-1 infection: A gp41 peptide induces the expression of an NKp44 ligand. Proc. Natl. Acad. Sci..

[66] Hessell A.J., Haigwood N.L. (2015). Animal models in HIV-1 protection and therapy. Curr. Opin. HIV AIDS.

[67] Mayr L.M., Su B., Moog C. (2017). Non-Neutralizing Antibodies Directed against HIV and Their Functions. Front. Immunol..

